# Contribution of FBLN5 to Unstable Plaques in Carotid Atherosclerosis *via* mir128 and mir532–3p Based on Bioinformatics Prediction and Validation

**DOI:** 10.3389/fgene.2022.821650

**Published:** 2022-03-09

**Authors:** Lin Zheng, Xinyang Yue, Minhui Li, Jie Hu, Bojin Zhang, Ruijing Zhang, Guoping Zheng, Ruihan Chen, Honglin Dong

**Affiliations:** ^1^ Department of Vascular Surgery, The Second Hospital of Shanxi Medical University, Taiyuan, China; ^2^ Department of Nephrology, The Second Hospital of Shanxi Medical University, Taiyuan, China; ^3^ The Second Hospital of Shanxi Medical University, Taiyuan, China; ^4^ Shanxi Medical University, Taiyuan, China

**Keywords:** carotid artery diseases, gene expression profiling, microarray analysis, biomarker, bioinformatics

## Abstract

FBLN5, a member of the short fibulins in the fibulin family of extracellular matrix/matricellular proteins, is involved in interactions with components of the basement membrane and extracellular matrix proteins. It plays key roles in endothelial tissues in many vascular diseases. In this study, the relationship between FBLN5 and carotid atherosclerotic plaque stability as well as the regulatory roles of miRNAs were evaluated. Differential gene expression analyses and weighted gene co-expression network analysis (WGCNA) based on the GSE163154 dataset (including 16 samples without intraplaque hemorrhage and 27 samples with intraplaque hemorrhage) in GEO revealed that FBLN5 is related to plaque stability and is the most significantly differentially expressed gene. LASSO regression was used to evaluate genes obtained from the intersection of differentially expressed genes and clinically significant modules identified by WGCNA. A prediction model based on eight genes, including *FBLN5,* was constructed and showed an accuracy of 0.951 based on an ROC analysis. Low FBLN5 expression in plaque tissues was confirmed by immunohistochemistry and western blotting. GO (Gene Ontology) and KEGG (Kyoto Encyclopedia of Genes and Genomes) enrichment analyses showed that FBLN5 acted mainly by the maintenance of the cellular matrix and reactive oxygen species production. miRNAs upstream of these eight predictive genes, including *FBLN5*, were identified and used to construct a network diagram. These results revealed that hsa-mir-128 and hsa-mir-532–3p were upstream regulatory factors of FBLN5, as verified by PCR assays of human plaque tissues demonstrating that both miRNAs were significantly up-regulated. Therefore, FBLN5 may play an important role in carotid atherosclerosis *via* hsa-mir-128 and hsa-mir-532–3p as well as become an essential target for treatment.

## Introduction

Stroke is a serious health issue. Carotid atherosclerosis is responsible for more than a third of strokes. However, little is known about the factors that cause atherosclerotic plaque instability, rupture, and embolism. Therefore, it is critical to clarify the molecular mechanism underlying the development and progression of intraplaque hemorrhage as well as to identify new molecular targets for pharmacological therapy to decrease plaque instability and avoid ischemic events.

McGeachie et al. (2009) constructed a Bayesian model to predict atherosclerosis based on 13 genes, including *FBLN5,* and 5 clinical variables and obtained a prediction accuracy of 85%; however, the mechanism by which the genes contribute to atherosclerosis was not explored. Colige et al. (2019) found that FBLN5 is connected to FBLN1 *via* LTBP4, and ADAMTST degrades LTBP4 in cardiovascular diseases, ultimately resulting in extracellular matrix degradation and disease development. In arterial dissection, Nox1 may negatively regulate FBLN5 to degrade the arterial middle layer (Hu et al., 2019). Carotid atherosclerosis also involves changes in the middle layer of the artery.

In this study, we explored the expression patterns and regulation of FBLN5 by a comparative analysis of available data for samples with stable and unstable carotid atherosclerotic plaques. In comparison with histologically stable plaques, we expected histologically unstable plaques to exhibit distinct gene and miRNA expression profiles. By a weighted correlation network analysis (WGCNA) and LASSO regression, we evaluated correlations between FBLN5 and clinical parameters. We validated our results in human and rat samples by western blotting and immunohistochemical assays. Furthermore, we validated miRNA expression levels by PCR. These findings reveal effective targets and upstream regulators for the prevention of intraplaque hemorrhage (IPH) and lay a foundation for the development of molecular prevention strategies for stroke onset (Langfelder and Horvath, 2008; Reid and Tibshirani, 2014).

## Materials and Methods

### Datasets

Carotid atherosclerosis gene expression profiles were obtained from the GSE163154 dataset in the Gene Expression Omnibus (GEO) database (http://www.ncbi.nlm.nih.gov/geo/). The series matrix file and platform data tables (GPL6104) were downloaded.

### Differential Gene Expression Analysis

The probe names in the matrix files were replaced with gene symbols, and GSE163154 was annotated with the GPL6104 platform data tables. Data were obtained for 43 samples, including 27 IPH samples and 16 samples lacking IPH. The presence of IPH indicated plaque instability. The limma package in R was used to find differentially expressed genes (DEGs) (Ritchie et al., 2015). Values of |log2 (fold change)| > 2 and adjusted-P < 0.05 were set as the thresholds for DEG screening. An advanced volcano plot was generated using the OmicStudio tools at https://www.omicstudio.cn/tool. A clustering analysis of DEGs was performed by using heatmap tools in Hiplot (https://hiplot.com.cn), a comprehensive web platform for scientific data visualization (Openbiox community, 2021).

### Construction of a Co-Expression Network

Based on the expression profile of GSE163154, the WGCNA package in R was used to design a co-expression network. The impute package in R was used to inspect the microarray data quality based on missing data and sample quality. A weighted gene co-expression network analysis (WGCNA) was performed with the top 20% of genes in the GSE163154 dataset. To visualize the sample tree and identify outliers, sample clustering was used. The soft thresholding power (β) value was calculated using the pickSoftTreshold function of WGCNA after computing Pearson’s correlation matrices for paired genes.

### Gene Ontology and Kyoto Encyclopedia of Genes and Genomes Analysis

GO enrichment and KEGG pathway analyses of the intersection between genes in the hub modules and DEGs were performed using the clusterProfiler package (for enrichment analyses) and org.hs.eg. db package (for ID conversion) in R 3.6.3, a search tool for the retrieval of interacting genes/proteins (Yu et al., 2012).

### Identification of Hub Genes

To identify novel genes related to intracellular signaling and alterations in transcription, a Cox regression analysis was carried out using expression levels of screened genes and the presence or absence of IPH. DEGs with a value of *p* < 0.05 were then evaluated by a univariate Cox proportional hazard regression analysis. The glmnet package in R was then used for a least absolute shrinkage and selection operator (LASSO) Cox regression analysis to find the genetic model with the best prognostic value. Finally, a prognostic signature for IPH was created by a multivariate Cox regression analysis. The expression of differentially expressed inflammatory response-related genes and the regression coefficients obtained in the regression model were used to compute the risk score for each patient. To calculate the risk score for each patient, the regression coefficient for each gene was multiplied by the expression level and the sum was obtained. The formula is as follows:

$$\mathrm{Risk}\;\mathrm{score}\left(\mathrm{patients}\right)=\sum_{i=1}^{n}\text{coefficient}\left(\text{gene}i\right)\times \text{expression}\;\text{value}\left(\text{gene}i\right)$$ (1)

Gene*i* is the *i*th gene and coefficient (gene*i*) is the estimated regression coefficient from the Cox proportional hazards regression analysis for gene *i*. The accuracy of the prognostic prediction model was evaluated using time-dependent ROC curves (Cole et al., 1991). GSE100927 downloaded from GEO contained the expression profile data for the carotid atherosclerotic group and the normal group, and R was used for analyses and visualization. In an ROC curve analysis, AUC of >0.60 indicated good predictive ability, and an AUC of >0.75 indicated high predictive value.

### Identification of Key miRNAs

Diagnostic genes predicted by LASSO were imported into starBase V3.0 to retrieve upstream microRNAs. GSE11794 was downloaded from the GEO database to obtain the microRNA expression profiles of symptomatic and asymptomatic patients with carotid atherosclerosis. All patients presented with significant carotid stenosis (60–100%). However, only symptomatic patients suffered a transient ischemic attack, transient monocular blindness ipsilateral to the study artery, or minor or non-disabling ipsilateral stroke. With data from GEO2R, ggplot2 package, OmicStudioclassic package, and OMicStudioKits package in R version 3.6.1 were used to screen differentially expressed genes. The significant thresholds were set to *p* < 0.05 and $\log_{2}\left|FC\right|\geq 1$ . An advanced volcano plot was generated using OmicStudio tools at https://www.omicstudio.cn/tool. The intersection between upstream miRNAs predicted by LASSO and differentially expressed miRNAs (DEMs) were identified as the key miRNAs related to carotid plaque stability. Cytoscape V3.7.2 was used to map the network interactions between genes and key miRNAs. The matrix of differently expressed hub genes in GSE163154 was obtained using OmicShare tools, a free online platform for data analysis (https://www.omicshare.com/tools).

### *In Vivo* Analyses

Three rats subjected to carotid ligation were starved for 14 h after the administration of a high-fat diet for 16 weeks and then sedated and killed. Another three rats were fed a normal diet. For the western blot analysis, the carotid tissues were extracted and snap frozen in liquid nitrogen.

### Specimens and Patients

Carotid samples (*n* = 3) from patients with carotid atherosclerosis who underwent carotid endarterectomy were obtained from the Second Affiliated Hospital of Shanxi Medical University. Each sample was divided into the part close to the plaque (disease group) and the part far from the plaque (normal group). Samples were used after obtaining informed written permission from patients. All arterial samples were collected following procedures authorized by the Office for Human Subjects Protection at Shanxi Medical University’s Second Affiliated Hospital.

### Western Blotting

Proteins were extracted from carotid tissues from rat and human tissue samples. Protein concentrations were measured by the BCA assay (Beyotime, China). Briefly, 25 μg of lysate samples were separated on NuPage 4–12% Bis-Tris Gels (Novex, Life Technologies, Carlsbad, CA, United States). The primary antibody against FBLN5 (1:200; ab202977) was purchased from Abcam (Cambridge, UK).

### Immunohistochemistry

Formalin-fixed and paraffin-embedded human carotid plaque tissue specimens were used for the immunohistochemical analysis. Carotid plaque tissues were obtained from the Second Affiliated Hospital of Shanxi Medical College. Three patients with carotid atherosclerosis were enrolled in this study. A 1:400 dilution of anti-FBLN5 (Abcam) was used as the primary antibody. Immunohistochemical results were evaluated by mean density. At least three 200× fields were randomly selected for each slide in each group. Effort was made to ensure that the field of vision was largely filled with tissue and each image had the same background light. Image-pro Plus 6.0 was used to select the same brown-yellow color as the unified standard to judge the positivity. The cumulative optical density (IOD) and pixel AREA (AREA) were obtained and IOD/AREA (mean density) was calculated.

### miRNA Isolation and Quantification by qRT PCR

Total RNA was isolated from plasma samples using RNA Rapid Extraction Solution (Servicebio, Wuhan, China), according to the protocol of the manufacturer. The quality of isolated RNA was evaluated using a NanoDrop spectrophotometer (Thermo Fisher Scientific, Waltham, MA, United States). Isolated RNA was reverse transcribed into cDNA using the Servicebio RT First Strand cDNA Synthesis Kit. Real-time PCR quantification of miRs was performed using sequence-specific 2× SYBR Green qPCR Master Mix (No ROX). The miR expression levels were normalized to the level of U6 small nuclear RNA and relative expression was estimated using the ΔΔCt method.

## Results

### FBLN5 Expression Differed Substantially Between Stable and Unstable Carotid Plaque Tissue Samples and was Correlated With Clinical Outcomes

**FIGURE 1:**
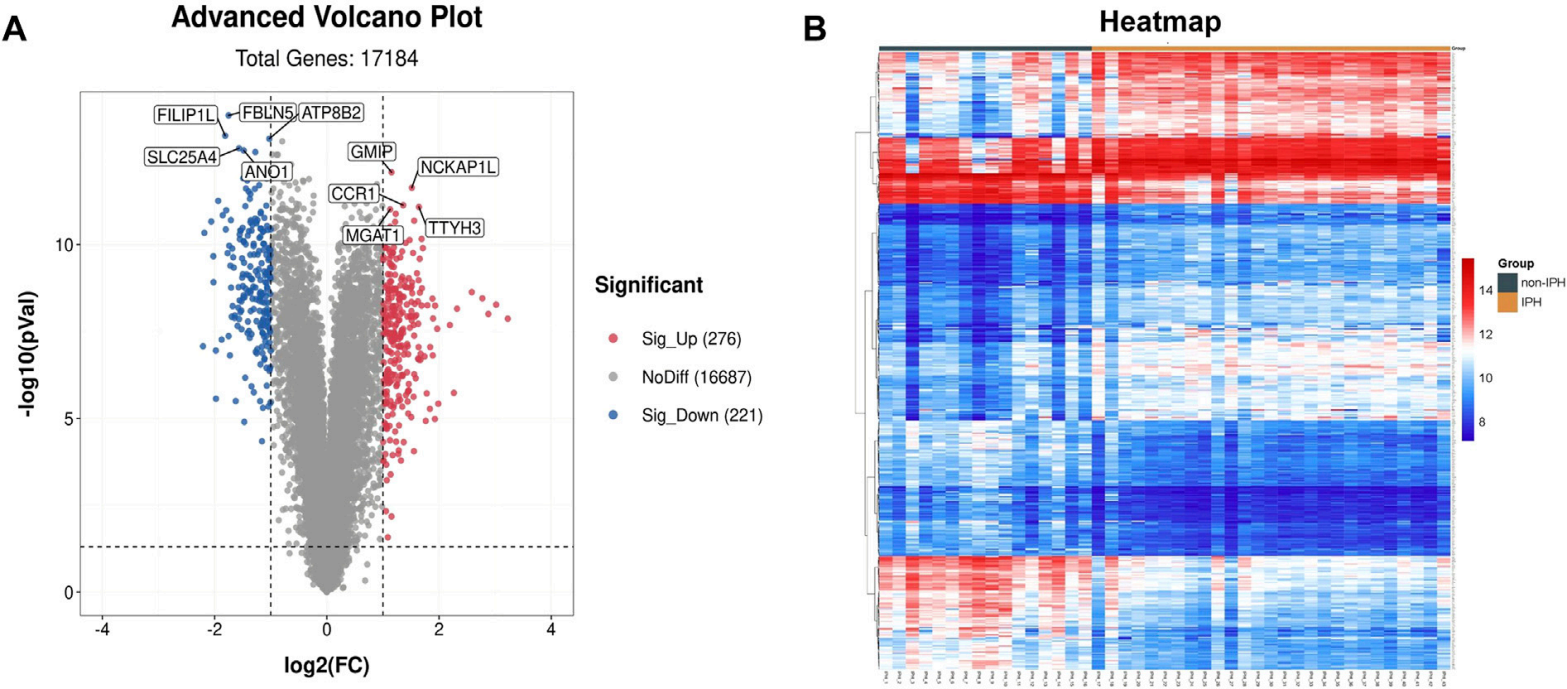
Identification of genes associated with carotid atherosclerosis. **(A)** Volcano plot of the DEGs. Red, upregulation; blue, downregulation. **(B)** Heatmap of DEGs.

To test whether FBLN5 plays a role in carotid atherosclerotic plaque stability, we download an expression matrix from a GEO dataset including 27 samples with IPH and 16 samples without IPH. The samples were carotid atherosclerotic plaques obtained from carotid endarterectomy surgery and included 16 stable atherosclerotic lesion segments and 27 unstable segments based on the presence of intraplaque bleeding. A total of 497 DEGs (276 upregulated and 221 downregulated in unstable plaques) were chosen for further investigation (using FDR <0.05 and fold change >2 as thresholds). A volcano plot and heatmap of the DEGs are shown in Figures 1A,B. We identified the five most highly up-regulated and down-regulated genes. *FBLN5* was down-regulated and, of note, was the most significant DEG in the dataset.

**FIGURE 2:**
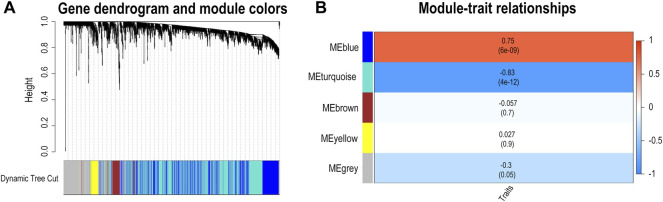
Identification of genes associated with carotid atherosclerosis. **(A)** Dendrogram based on a dissimilarity metric for all differentially expressed genes (1-TOM). **(B)** Relationship between module eigengenes and intraplaque bleeding as a heatmap.

**FIGURE 3:**
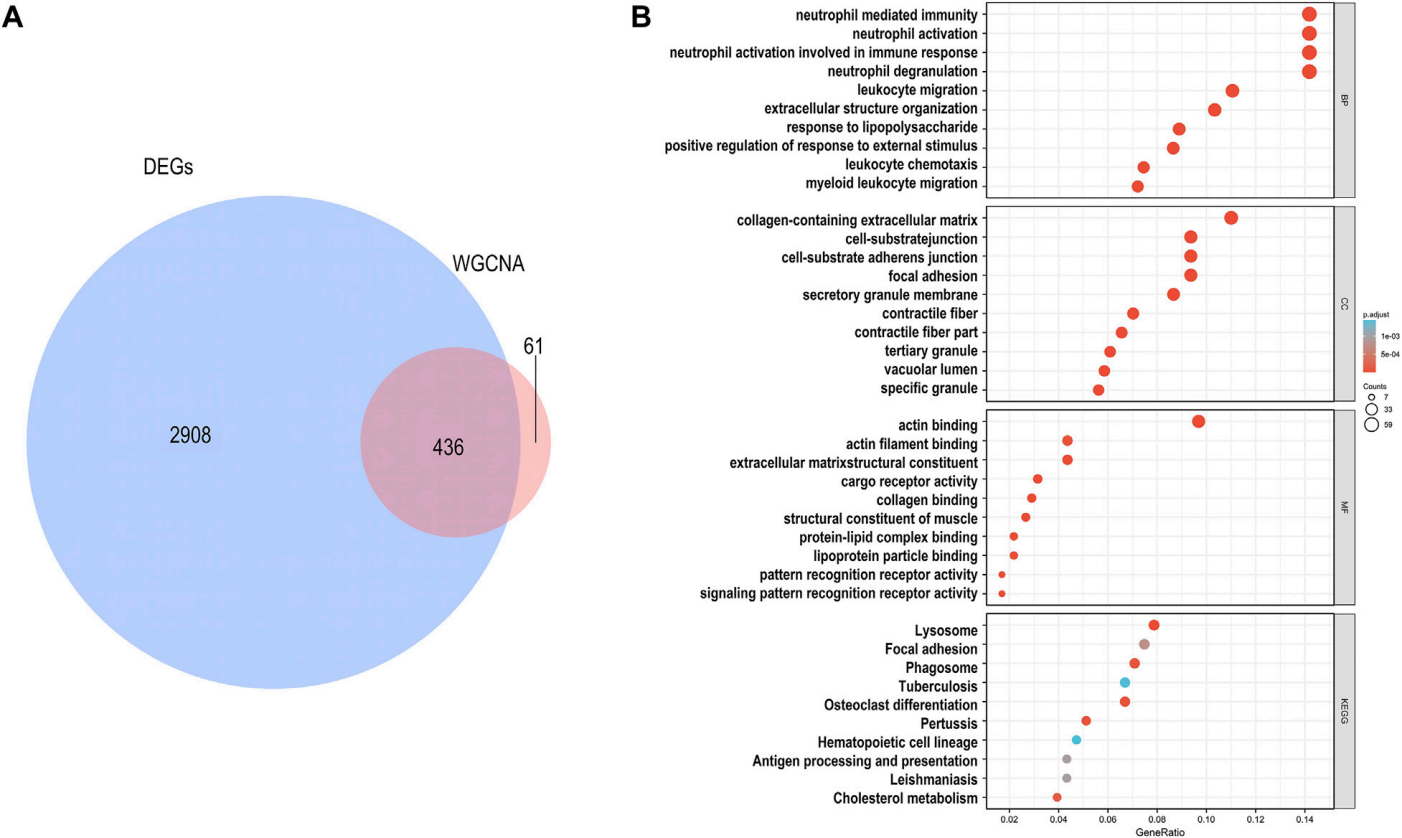
Identification of genes associated with carotid atherosclerosis. **(A)** Intersection of DEGs and module genes associated with clinical parameters. **(B)** The top 10 pathways of in the GO enrichment analysis (BP, MF, and CC) and KEGG pathway analysis. FDR q-value < 0.05.

To further verify whether differences in *FBLN5* were related to clinical outcomes, namely carotid plaque stability, we performed a WGCNA and generated a LASSO prediction model. We matched the illness states to the expression matrices of samples. A sample dendrogram and trait heatmap were generated after the 43 samples were assigned to groups, as shown in Supplementary File S1. To design a weighted network based on a scale-free topology, the soft thresholding power was set to 17, where the curve first approached *R* ^2^ = 0.85 (Supplementary File S2). The dynamic tree cutting method was used to identify five modules, as shown in Figure 2A. High correlations were found with the disease status (IPH or non IPH) after linking the modules to traits, as illustrated in Figure 2B. The blue and turquoise modules were identified as clinically significant (*p* < 0.05) and used for further investigation. *FBLN5* was assigned to modules showing significant downregulation. The intersection of DEGs and genes in two clinical modules included 436 genes, as shown in Figure 3A. Figure 3B lists the enriched GO terms and KEGG pathways. The common hub genes were significantly enriched in the following terms in the GO biological processes (BP) category: neutrophil mediated immunity, neutrophil activation, neutrophil activation involved in immune response, and extracellular structure organization. Enriched pathways mainly included extracellular structure organization, extracellular matrix organization, regulation of reactive oxygen species metabolic process, reactive oxygen species metabolic process cellular substrate adhesion, cell-matrix adhesion, superoxide metabolic process, response to reactive oxygen species, and response to oxidative stress. These findings indicate that carotid plaque stability is mainly related to the immune response, with a key role of neutrophils. FBLN5 is mainly responsible for the maintenance of the cellular matrix and reactive oxygen species production (Supplementary File S3). Extracellular superoxide dismutase protects the arterial endothelium by binding to FBLN5 to reduce superoxide anion (O_2_^*-^) levels in atherosclerosis (Nguyen et al., 2004). The enrichment analysis and previous results reveal that *FBLN5* may also play a role in reducing O_2_^*-^ in carotid atherosclerosis.

**FIGURE 4:**
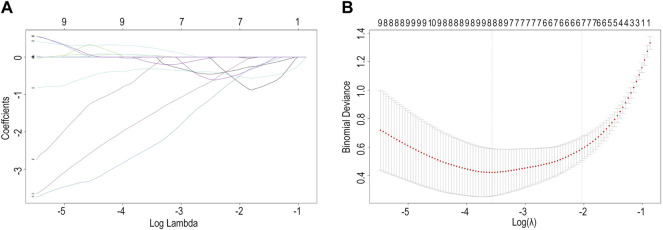
**(A)** Ridge trace. **(B)** Cross-validation score.

**FIGURE 5:**
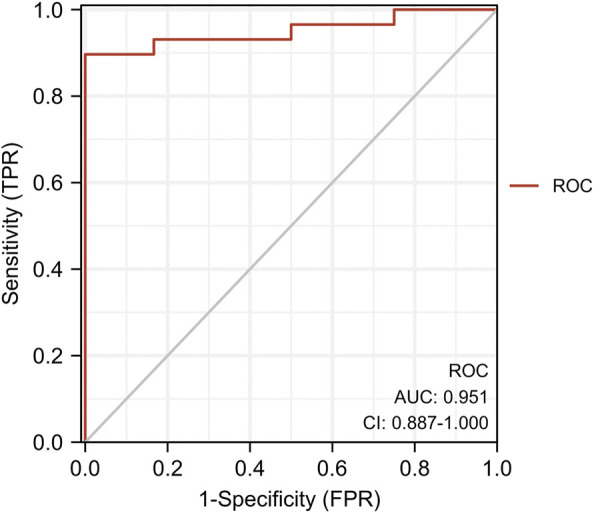
ROC analysis of target gene co-expression in the training set.

We found eight optimal prognostic genes by LASSO regression and included these genes in a prognostic risk model: *FBLN5, FMOD, GAL, GEM, SLC14A1, SPTBN1, TMEM119,* and *GREM1* (Figures 4A,B). We calculated the risk score for each patient using mRNA levels and risk regression coefficients to identify the relevance of hub genes (see Material and Methods section for the formula) as follows: Risk score = (−0.31774005 × expression of *FBLN5*) + (−1.58406823 × expression of *FMOD*) + (0.04517572 × expression of *GAL*) + (−2.44920986 × expression of *GEM*) + (0.04109032 × expression of *SLC14A1*) + (−0.12978159 × expression of *SPTBN1*) + (−0.33417587 × expression of *TMEM119*) + (0.25663856 × expression of *GREM1*). An ROC curve analysis was performed to evaluate the diagnostic value of these prognostic genes in patients with ruptured or stable human atheromatous lesions and the AUC value for the overall model was 0.951 (Figure 5). *TMEM119* was not evaluated in the ROC curve analysis owing to the absence of data in this dataset. The mRNA levels of these genes accurately distinguished IPH tissues and the model had a high predictive accuracy. This demonstrates that the genes obtained by the LASSO regression analysis are potential diagnostic biomarkers for IPH. Therefore, *FBLN5* is not only associated with clinical outcomes but also has predictive value for carotid atherosclerotic plaque stability.

### Confirmation of Low FBLN5 Expression at the Protein Level in Human and Rat Samples

**FIGURE 6:**
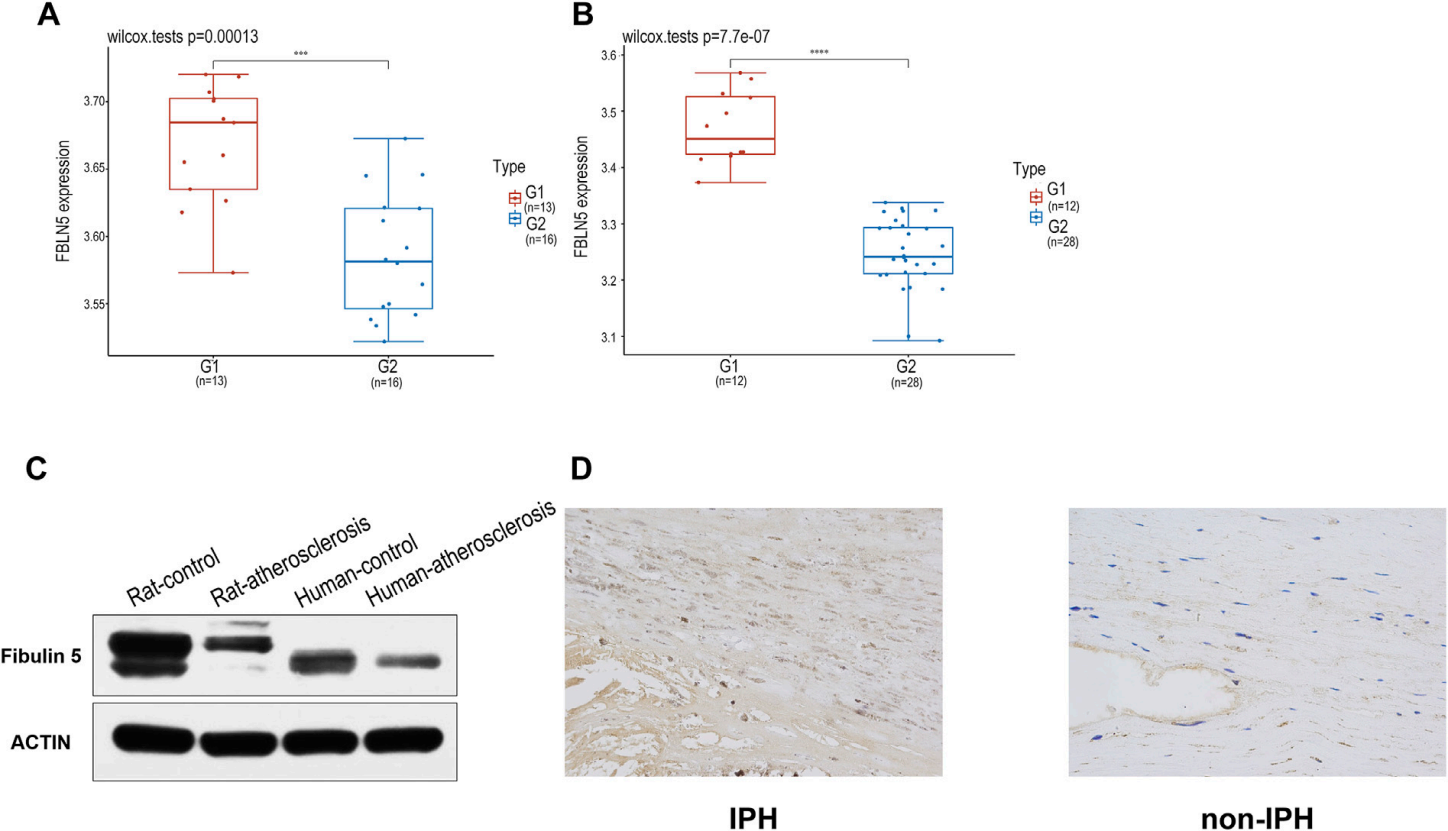
**(A, B)** Box plots of GSE100927 and GSE28829 after data standardization. Blue represents different disease sets, and red represents normal sets. **(C)** Decreased FBLN5 expression in carotid atherosclerosis tissues was detected by western blotting. **(D)** Representative images of FBLN5 immunohistochemistry in carotid tissue.

Using GSE28829 and GSE100927 datasets, after data standardization, FBLN5 was the most significantly differentially expressed locus between IPH and non-IPH (Figures 6A,B). To validate the expression of FBLN5 in advanced plaque formation, we constructed a rat model by feeding rats subjected to carotid artery ligation a high-fat diet for 16 weeks. We detected the level of FBLN5 in human carotid plaque tissues by western blotting, indicating that FBLN5 was significantly down-regulated in plaque compared with non-plaque artery tissues (Figure 6C). Immunohistochemical staining results showed that the mean density of carotid plaques was 0.0009 ± 0.0001 and that of the control group was 0.0156 ± 0.00359, showing that FBLN5 was more highly expressed in the normal tissues (Figure 6D), and this difference was significant (*p* < 0.001) (Supplementary File S4).

### Analysis and Identification of Hsa-miR-128 and Hsa-miR-532–3p as Upstream Regulators of FBLN5

**FIGURE 7:**
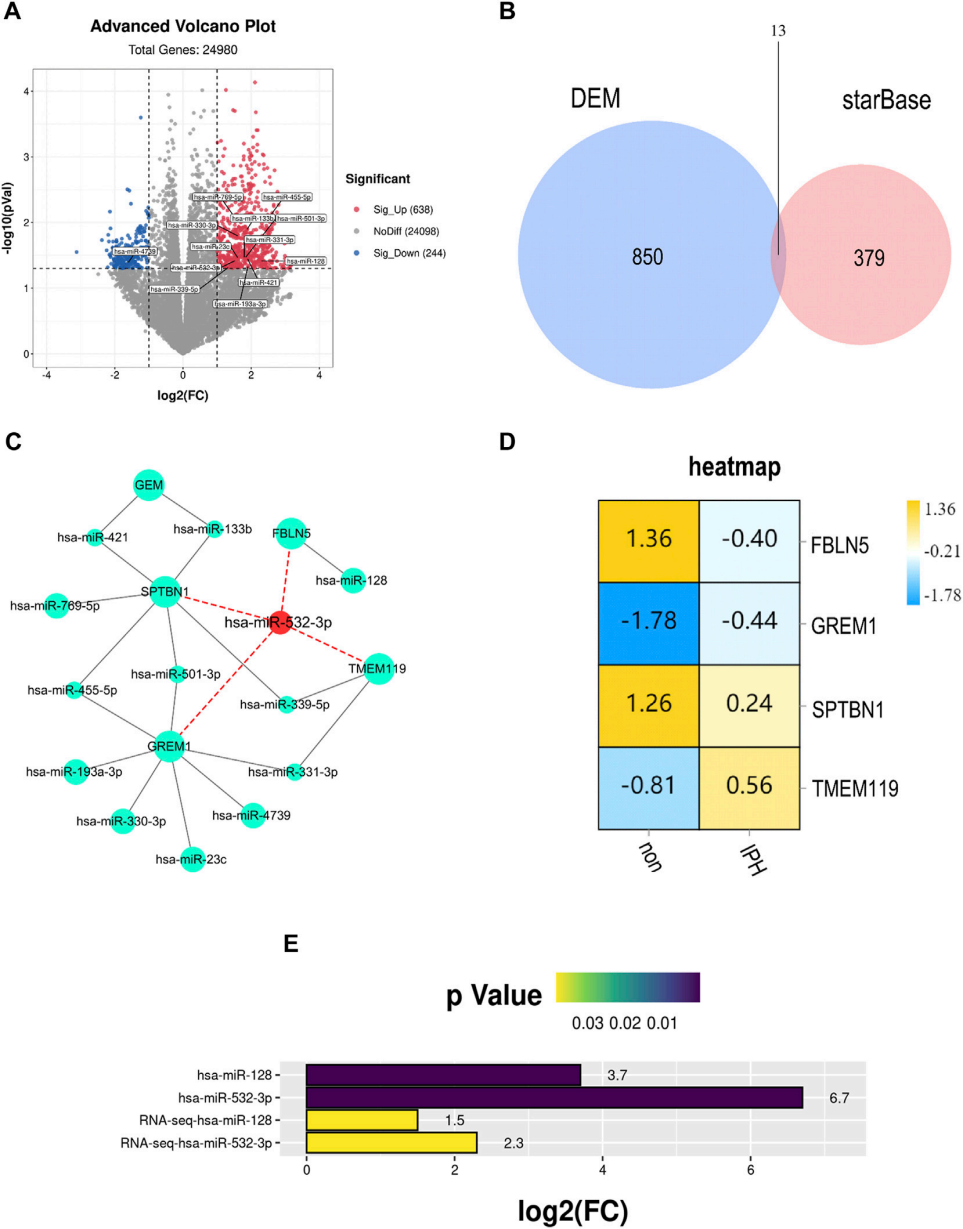
**(A)** Volcano plot of DEMs. Red, upregulation; blue, downregulation. Thirteen genes related to plaque rupture were identified. Except for hsa-miR-4739 downregulation, other miRNAs were significantly up-regulated. **(B)** Intersection of DEMs and upstream microRNAs of hub genes. There are 13 overlapping miRNAs. **(C)** Network of intersection miRNAs and hub genes. hsa-miR-532–3p is the most core miRNA, which has connections with hub genes. **(D)** Decreased FBLN5 expression in carotid atherosclerosis tissues was detected by western blotting. **(E)** P and $\log_{2}\left|FC\right|$ of PCR detection and RNA-seq expression of the two miRNAs upstream of FBLN5 in the stable group and the unstable group.

We identified possible up-regulated microRNAs associated with FBLN5 after confirming that FBLN5 expression is reduced in unstable samples of carotid atherosclerosis. We searched for miRNAs upstream of eight hub genes in starBase Version3.0. GSE11794 (an ncRNA dataset for carotid atherosclerosis) was downloaded for a differential expression analysis. In total, 638 up-regulated and 244 down-regulated ncRNAs were obtained (*p* < 0.05, $\log_{2}\left|FC\right|\geq 1$ ), and the volcano plot is shown in (Figure 7A). The intersection of DEMs with upstream microRNAs revealed 13 core upstream regulators (Figure 7B), including hsa-miR-769–5p, hsa-miR-532–3p, hsa-miR-501–3p, hsa-miR-4739, hsa-miR-455–5p, hsa-miR-421, hsa-miR-339–5p, hsa-miR-331–3p, hsa-miR-330–3p, hsa-miR-23c, hsa-miR-193a-3p, hsa-miR-133b, and hsa-miR-128. These miRNAs and their closely related hub genes were introduced into Cytoscape to draw an interaction network (Figure 7C). miR-532–3p was linked to the largest number of hub genes. These findings suggested that mir-532–3p is closely related to the rupture of the carotid plaque. Among the intersecting genes, *FBLN5* expression differed significantly after plaque rupture (Figure 7D). We used human samples to validate the expression changes of hsa-miR-128 and hsa-miR-532–3p during advanced plaque formation. miRNA levels were significantly higher in the advanced plaque group than in the control group (*p* < 0.0001) (Figure 7E). In conclusion, FBLN5 may play a role in the progression of carotid atherosclerosis *via* negative regulatory effects of hsa-miR-128 and hsa-miR-532–3p.

## Discussion

Stroke is a deadly disease, and its mortality rate has been rising worldwide in recent years. Carotid atherosclerotic is an important cause of stroke. Although fat, smoking, and chronic diseases are considered risk factors, the etiology of unstable plaque in carotid atherosclerotic is unclear owing to conflicting results (Libby et al., 2019; Chen et al., 2020). Understanding the molecular mechanism underlying unstable plaque is of great importance for diagnosis and treatment. Numerous studies have defined the gene signatures that distinguish stable plaques from unstable ones, and many studies have confirmed that ncRNAs contribute to unstable plaque formation in carotid atherosclerosis by binding to and down-regulating mRNAs (Lucas et al., 2018; Fasolo et al., 2019; Ryu et al., 2021). In this study, we clearly establish *FBLN5* as a novel diagnostic biomarker and miRNA-532–3p and miRNA-128 as upstream regulators. Western blotting and immunohistochemistry further validated the downregulation of FBLN5 and upregulation of miRNA expression levels. Therefore, we suspect that FBLN5 may influence carotid atherosclerotic development *via* the regulation of miRNA-128 and miRNA-532–5p.

FBLN5 showed significant differences in unstable plaques throughout our data analysis. First, *FBLN5* was the most highly differentially expressed. Second, in a WGCNA, *FBLN5* was in the module that was significantly related to the stability of carotid atherosclerotic plaques. Finally, in a LASSO analysis, *FBLN5* was among eight genes in the prediction model, and the accuracy of the model was 0.951. *FBLN5* was an effective gene for predicting plaque stability. In the process of experimental verification, differences in the expression of FBLN5 between the two groups were demonstrated at both mRNA and protein levels. To determine the upstream regulatory mechanism, we predicted interacting miRNAs and obtained Hsa-mir-532–3p and Hsa-Mir-128. These miRNAs were up-regulated in a PCR verification experiment, indicating that they may regulate *FBLN5* in carotid atherosclerosis.

FBLN5 encodes a secreted extracellular matrix protein with an Arg-Gly-Asp (RGD) motif and calcium-binding EGF-like domains. Through interactions between integrins and the RGD motif, it enhances endothelial cell adhesion. It is highly expressed in arteries that are still growing. In balloon-injured arteries and atherosclerotic lesions, however, its expression is reduced, particularly in intimal vascular smooth muscle cells and endothelial cells. Numerous studies have confirmed that FBLN5 has predictive value for the outcome of coronary calcification (Spencer et al., 2005; Sullivan et al., 2007). FBLN5 directly interacts with elastic fibers through its amino-terminus and provides anchorage to stabilize and organize the vasculature (Nakamura et al., 2002). As a result, the protein may function in vascular development and remodeling. After ligation of the carotid artery, FBLN5 (−/−) animals show severe carotid intima hyperplasia, smooth muscle cell proliferation, and migration (Spencer et al., 2005). Our results demonstrate that in patients with carotid atherosclerosis, the expression levels of FBLN5 and anchoring protein in both smooth muscle cells and endothelial cells are reduced, resulting in weak intercellular interactions and increased cell proliferation and migration. As a regulator of FBLN5, miR-128 affects VSMC proliferation, migration, differentiation, and contractility by targeting Kruppel-like factor 4 and modulating the methylation status of the pivotal VSMC gene myosin heavy chain 11 (Myh11) (Farina et al., 2020). The CHROME lncRNA regulates cellular and systemic cholesterol homeostasis in atherosclerotic plaques by inhibiting miRNA-128–3p expression (Hennessy et al., 2019). miRNA-532 is associated with vulnerable plaques and modified low-density lipoprotein or tumor necrosis factor α exposure. The lncRNA CASC2 suppresses cell proliferation and promotes apoptosis by regulating the miR-532–3p/PAPD5 axis in ox-LDL-mediated VSMCs (Wang et al., 2020).

Our results provide novel molecular targets for predicting advanced plaque progression. However, additional factors need to be combined to predict adverse clinical outcomes. Additionally, the functional pathways of ncRNAs and FBLN5 should be further evaluated to develop effective strategies for preventing cardiovascular events.

## Conclusion

FNLN5 might be regulated by miRNA-532–3P and miRNA-128, providing potential targets for therapies and biomarkers for diagnosis and prognosis. Further studies of the underlying mechanisms are needed to explore the roles of these factors in the pathogenesis and progression of carotid atherosclerosis.

## Data Availability

Publicly available datasets were analyzed in this study. This data can be found here: GSE163154 https://www.ncbi.nlm.nih.gov/geo/query/acc.cgi?acc=GSE163154 GSE100927 https://www.ncbi.nlm.nih.gov/geo/query/acc.cgi?acc=GSE100927 GSE28829 https://www.ncbi.nlm.nih.gov/geo/query/acc.cgi?acc=GSE28829 GSE11794 https://www.ncbi.nlm.nih.gov/geo/query/acc.cgi?acc=GSE11794 and also in the Supplementary materials.

## Abbreviations

DEG, Differentially expressed gene; DEIRG, Differentially expressed inflammatory response-related gene; DEM, Differentially expressed miRNA; GEO, Gene Expression Omnibus; IPH, Intraplaque hemorrhage; KEGG, Kyoto Encyclopedia of Genes and Genomes; LASSO, Least absolute shrinkage and selection operator; ROC, Receiver operating characteristic; SMC, Smooth muscle cell; WGCNA, Weighted gene co-expression network analysis.
