# Supplementary figures and images for: Contribution of FBLN5 to Unstable Plaques in Carotid Atherosclerosis via mir128 and mir532–3p Based on Bioinformatics Prediction and Validation
Source: Front Genet. 2022 Mar 9;13:821650. doi: 10.3389/fgene.2022.821650 (PMC8959633; doi:10.3389/fgene.2022.821650)

**scale independence**

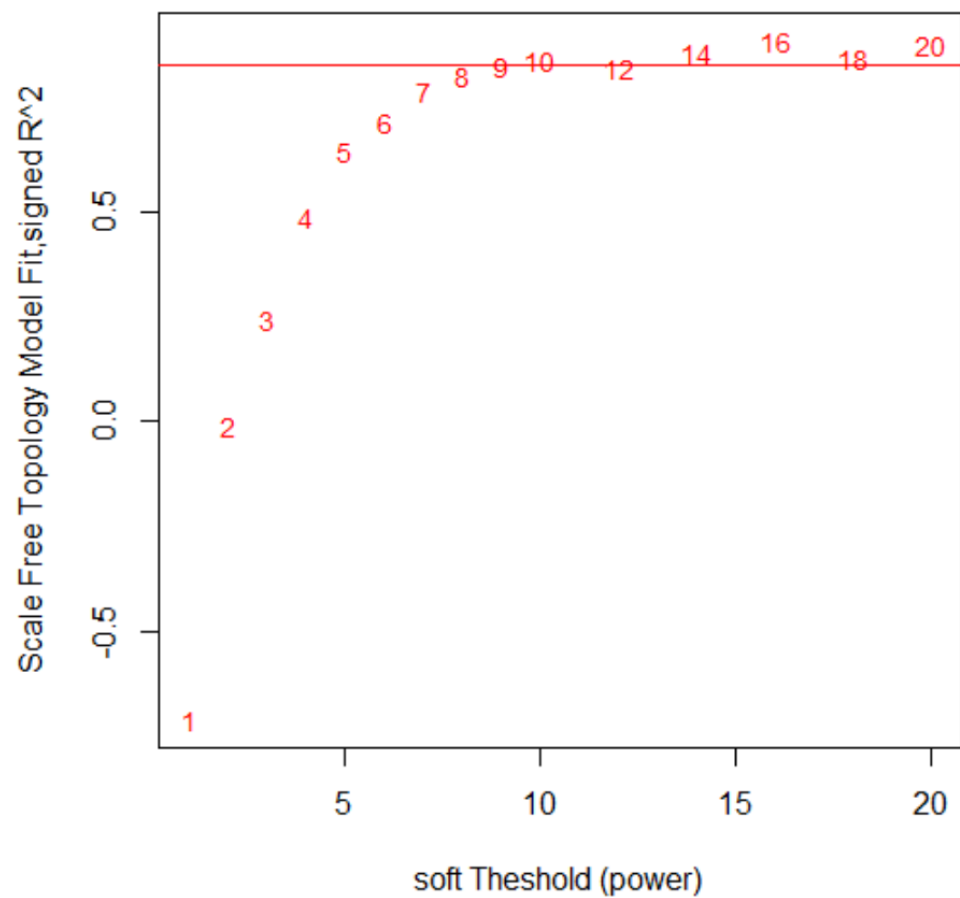

**Mean connectivity**

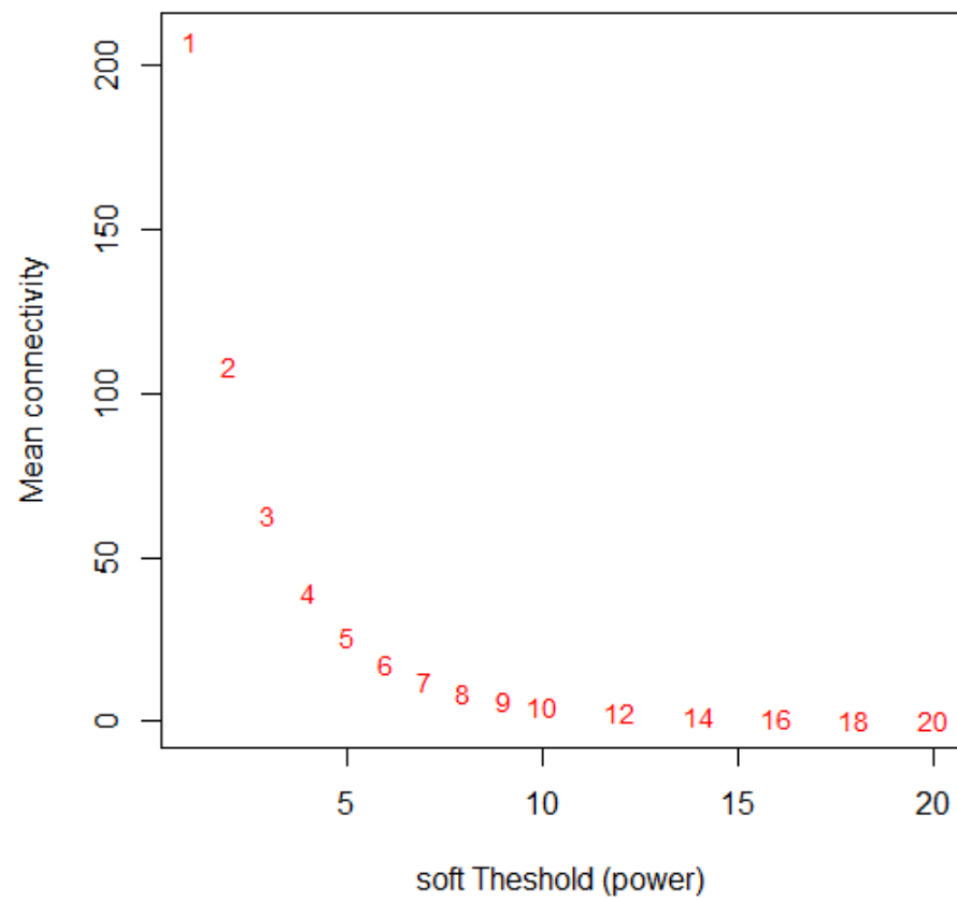

Supplement: Supplementary file 1 [file DataSheet2.PDF]

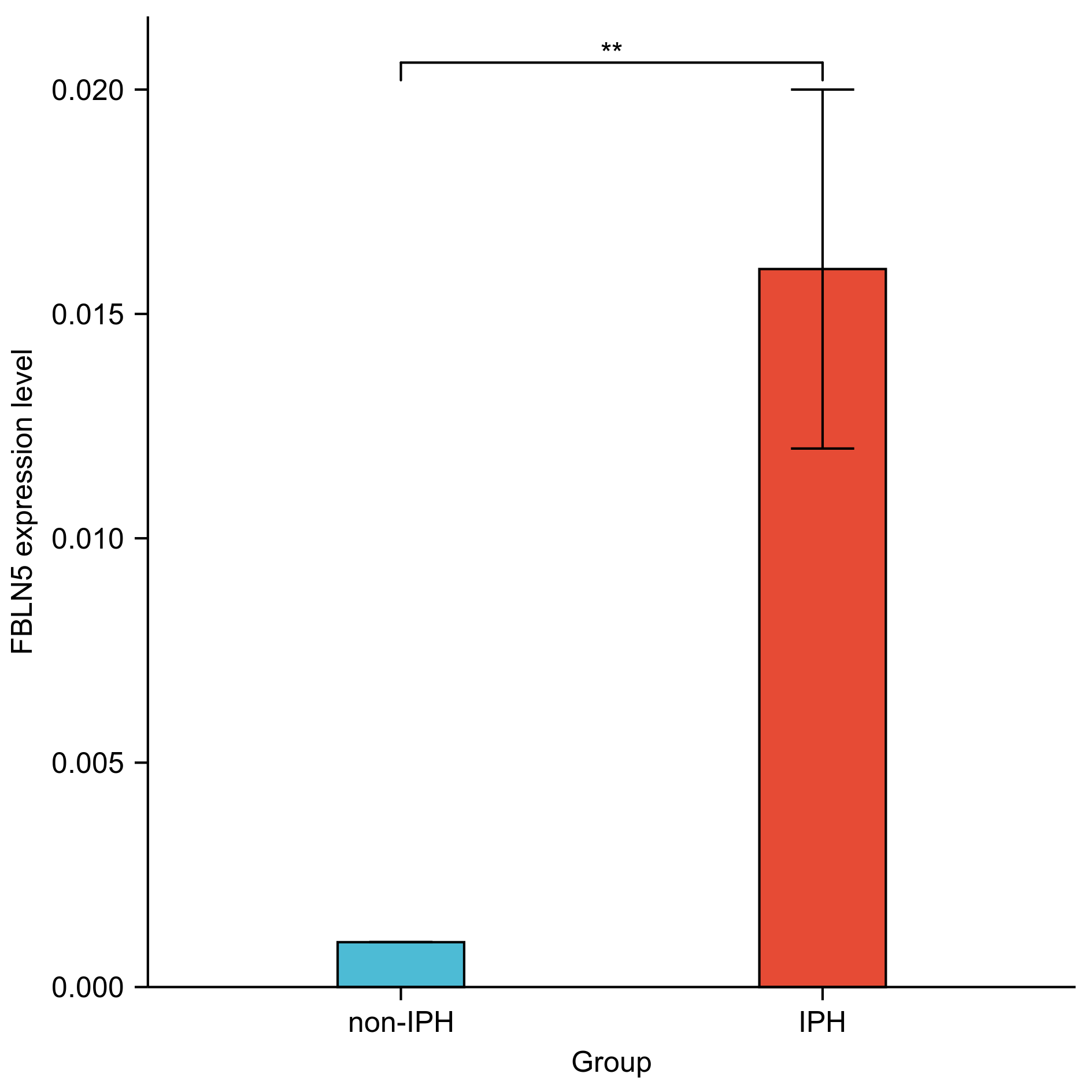

Supplement: Supplementary file 3 [file Image1.TIFF]

## sample clustering to detect outliers

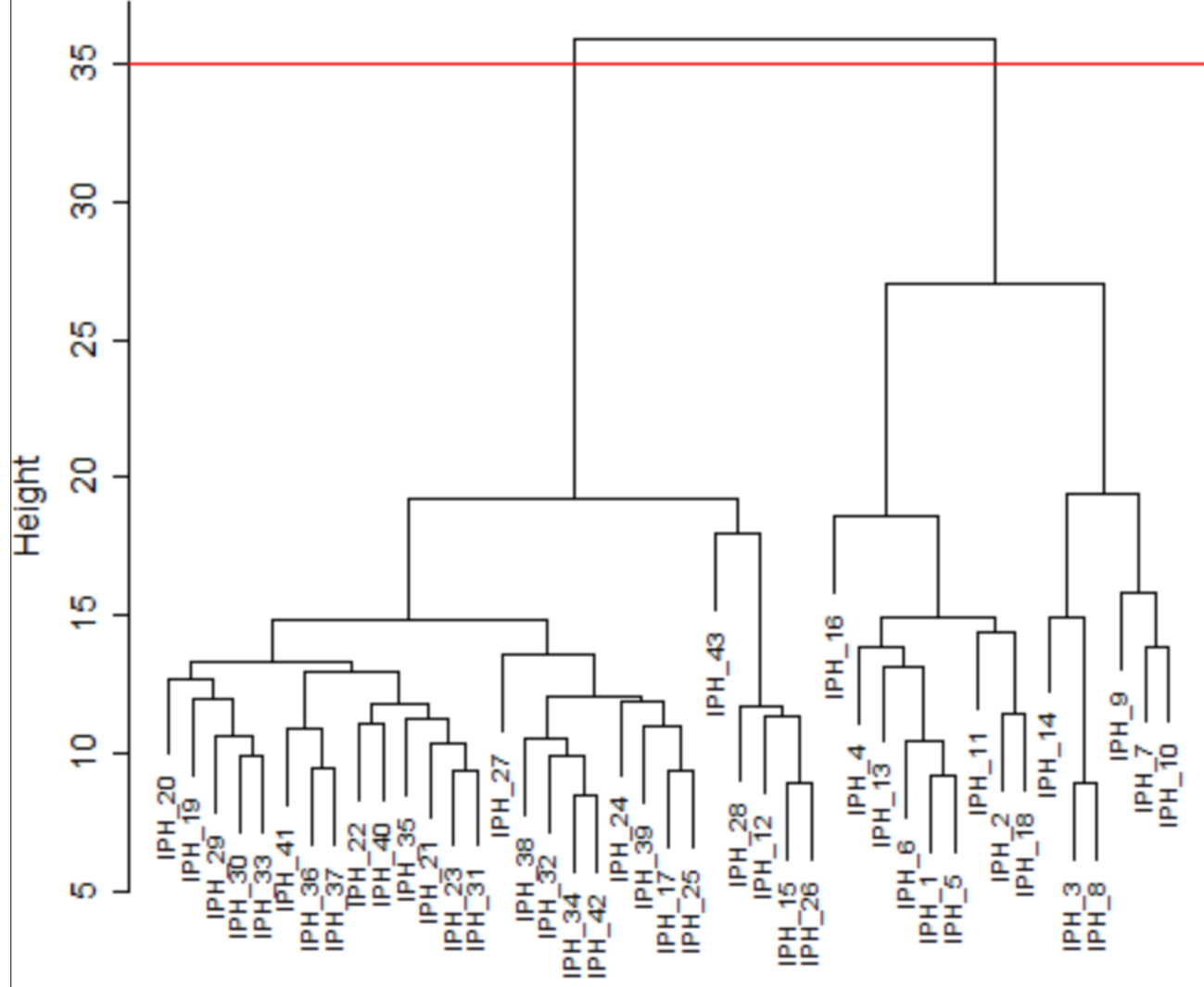

Supplement: Supplementary file 5 [file DataSheet1.PDF]
